# Residents of Central Queensland, Australia Are Aware of Healthy Eating Practices but Consume Unhealthy Diets

**DOI:** 10.3390/sports5040094

**Published:** 2017-12-11

**Authors:** Vincent J. Dalbo, Matthew I. Hiskens, Masaru Teramoto, Michael I. Kingsley, Kaelin C. Young, Aaron T. Scanlan

**Affiliations:** 1 Human Exercise and Training Laboratory, Central Queensland University, Rockhampton, Queensland 4701, Australia; a.scanlan@cqu.edu.au; 2 School of Health, Medical and Applied Sciences, Central Queensland University, Rockhampton, Queensland 4701, Australia; m.hiskens@cqu.edu.au; 3 Division of Physical Medicine and Rehabilitation, University of Utah, Salt Lake City, UT, 84108, USA; Masaru.Teramoto@hsc.utah.edu; 4 La Trobe Rural Health School, La Trobe University, Bendigo, Victoria 3550, Australia; M.Kingsley@latrobe.edu.au; 5 Edward Via College of Osteopathic Medicine, Auburn University, Auburn, AL, 36849, USA; kyoung@auburn.vcom.edu

**Keywords:** nutrition, knowledge, education

## Abstract

We aimed to determine nutritional knowledge and behaviors of normal weight, overweight, and obese residents of Central Queensland, Australia. Data were collected as part of the 2010 Central Queensland Social Survey (*N* = 1289). Residents were asked questions assessing nutritional knowledge and behaviors. Statistical analyses were performed to examine differences in nutritional knowledge and behaviors by body mass index (BMI) classification: normal weight, overweight, and obese. Independent of BMI, residents ate fewer than the recommended daily servings of vegetables (*p* < 0.05) and fruits (*p* < 0.05) with no differences found between BMI classifications. Overweight (OR: 1.52; 95% CI: 1.13–2.04) and obese (OR: 1.43; 95% CI: 1.04–1.98) residents were more likely to have eaten fast food the week of the survey than normal weight residents. Residents correctly identified the amount of kilocalories required to maintain current body weight with no differences between BMI classifications. Each BMI classification underestimated the amount of kilojoules required to maintain current body weight (*p* < 0.05). Nutritional knowledge may not be the limiting factor preventing residents from making proper nutritional choices.

## 1. Introduction

The concept of weight management is uncomplicated and founded on caloric balance, whereby weight gain is the result of excess caloric consumption compared to caloric expenditure. However, overweight/obesity has become a pandemic [1] and is a distinctly concerning health issue in Australia as 63% of adults and 25% of children are overweight/obese [2]. Further, the prevalence of overweight/obesity is continuing to rise [3]. Overweight/obesity presents significant fiscal challenges to healthcare systems [4] and substantial costs to the individual resulting in decreased quality and duration of life [5]. Specifically, overweight/obesity is associated with numerous health complications including metabolic syndrome, some cancers, and emotional health issues [6]. In Australia, healthcare costs are increasing at a faster rate than gross domestic product [7] with much expenditure being the result of overweight/obesity ($8.6 billion in 2011–2012; costs presented in 2014–2015 dollars) [8].

There appears to be consensus that increasing nutritional knowledge is required to improve nutritional behaviors [9,10,11,12,13]. In this regard, a review by Barbosa et al. [10] reported nutritional knowledge to be positively associated with several aspects of diet in adults including healthy eating, increased vegetable and fruit consumption, and a low intake of simple sugars, fat, and salt. However, in the same review, nutritional knowledge was not found to be associated with body mass index (BMI). Of the manuscripts reviewed, only 43% of studies reported an association between BMI and nutritional knowledge with positive and negative associations observed [10]. Further, articles included in the review by Barbosa et al. occurred in varied populations across numerous countries. In Australia, O’Dea and Wilson [14] found nutritional knowledge was not associated with BMI in 4441 students ranging in age from 6–18 years. More recently, Hendrie et al. [15] examined the demographic variation in nutritional knowledge in a convenience sample of 201 residents of Adelaide, South Australia. The primary finding from Hendrie et al. [15] was key dietary guidelines such as eating more fruit and vegetables were acknowledged by the community but detailed knowledge of nutrient content in foods and converting nutritional knowledge to food choice was poor. Given the belief that increasing nutritional knowledge is required to improve nutritional behaviors and the conflicting evidence suggesting nutritional knowledge does not appear to be the cause of overweight/obesity [14,16,17,18,19], it is important to determine nutritional knowledge and behaviors of normal weight, overweight, and obese adults residing in Australia.

## 2. Materials and Methods

### 2.1. Pilot Testing

Ethical clearance was granted by the Central Queensland University Human Research Ethics Committee (project number: H09/08-046; approval date: 2009) prior to data collection. All procedures were conducted in accordance with the Declaration of Helsinki. Pilot testing on 50 randomly selected households was conducted to examine interview comments (e.g., inadequate response categories, confusing wording, and question-order effects) and pre-test frequency distributions were reviewed to ensure questions did not require modification. Respondents were randomly drawn from a telephone database for Central Queensland using a ten-station computer-assisted telephone interviewing system (Ci3 CATI System, Win Cati 4.2, Sawthooth Technologies, Northbrook, IL, USA) installed on a local area network. Following pilot testing, all questions were deemed appropriate for inclusion in the survey. Data obtained during pilot testing were not included as part of the survey results. 

### 2.2. Survey Methods

Data were collected in October and November 2010 as part of the Central Queensland Social Survey [7]. Central Queensland was defined as the area from the Mackay Statistical Division to the northern point of the Wide Bay Burnett Statistical Division including Bundaberg. The region of Central Queensland was delineated into three areas for interviewing: Rockhampton Regional Council Area, Mackay Regional Council Area, and the remainder of Central Queensland. A random selection process was employed to ensure respondents had an equal chance of being selected using a two-stage process: (1) the selection of households; and (2) the selection of respondent gender in each household. One person was selected as the respondent for the interview per household. Respondents were 18 years or older and were living in a dwelling unit that could be contacted by a direct-dialed, land-based telephone service. If interviewers were unsuccessful establishing contact on their first call, a minimum of five call back attempts were made. Interview times complied with the revised Australian Communications and Media Authority Industry Standard for research calls.

### 2.3. Survey Respondents

Respondents completed a computer-assisted telephone interview representing 1289 completed interviews. The response rate of 36% is consistent with previous surveys from Central Queensland [20]. The Index of Dissimilarity demonstrated the sample collected during the survey varied from the target population according to Census data [2] on the dimensions of geographic region and age. As a result, data were adjusted for sampling weights determined using a one-step multiple weighting for geographic location and age category which has previously been reported [7].

### 2.4. Survey Structure

The survey consisted of a standardized introduction, demographic questions, and questions related to nutritional and physical activity knowledge and behaviors. Specific questions to assess nutritional knowledge and behaviors can be found in Table 1. Physical activity was assessed using a valid [21] and reliable [22] instrument based on the Active Australia Survey. BMI was ascertained by asking respondents to report their height and weight. For the questions examining how much energy should be consumed per day to maintain current body weight, resting metabolic rate of each respondent was estimated using the Mifflin-St Jeor equation [23]. 

### 2.5. Statistical Analyses

Data analyses included univariate and multivariate techniques. Descriptive statistics were calculated for sociodemographic measures. Separate logistic regression analyses were performed to examine potential differences in nutritional knowledge and behaviors by BMI classification. When possible, results were examined using gender, age, and education as covariates as these variables have been found to influence nutritional knowledge in Australia [15]. All statistical analyses were conducted using IBM SPSS Statistics (v 22.0, IBM Corporation, Armonk, NY, USA). Statistical significance was set at *p* < 0.05.

### 2.6. Nutritional Knowledge

The ability to identify saturated fat as a poor food choice and protein as the correct food choice to build or maintain muscle is presented as the percentage of residents in each BMI classification who answered each question correctly. Odds ratios with 95% confidence intervals were then computed using separate binary logistic regression analyses with gender, age, and education as covariates to determine if differences existed between the reference group (normal weight) and overweight, or obese respondents. 

Residents in each BMI classification who monitor kilocalories or kilojoules are presented as a percentage. An odds ratio with 95% confidence intervals was computed using a binary logistic regression analyses with gender, age, and education as covariates to determine if differences existed between the reference group (normal weight) and overweight, or obese respondents. Due to the low response rates and large differences in sample sizes across the BMI classifications, we used nonparametric statistical tests to examine the response pertaining to how many kilocalories and kilojoules each respondent should consume to maintain their current body weight relative to each BMI classification. Specifically, separate Kruskal-Wallis tests with Monte Carlo simulations were used to examine differences in responses by BMI classification regarding kilocalories and kilojoules. Furthermore, separate one-sample Wilcoxon signed-rank tests were used to examine if median kilocalories or kilojoules reported from any of the BMI classifications were different than the calculated values of 1600 kilocalories and 7000 kilojoules required by the residents to maintain current body weight.

### 2.7. Nutritional Behaviors

Self-reported servings of vegetables and fruits consumed per day are expressed as means ± 95% confidence intervals for each BMI classification. Separate, one-way analyses of variance (ANOVA) were performed to compare vegetable and fruit consumption between BMI classifications. Results were also examined using separate linear regression analyses to ensure the ANOVA results held true after adjusting the results for the covariates of gender, age, and education. Separate one-sample t-tests were computed to determine if residents in each BMI classification met the recommended consumption of vegetables and fruits. 

Residents in each BMI classification who ate fast food during the last week are presented as a percentage. An odds ratio with 95% confidence intervals was computed using a binary logistic regression analyses with gender, age, and education as covariates to determine if differences existed between the reference group (normal weight) and overweight, or obese respondents.

## 3. Results

### 3.1. Nutritional Knowledge

Sociodemographic information for the respondents is summarized in Table 2. Table 3 shows the responses to the questions assessing nutritional knowledge. Respondents were able to identify saturated fat as the poor food choice, with 72.8% of pooled respondents (i.e., percentage of correct responses out of all responses adjusted for sampling weights regardless of BMI classification) answering this question correctly. The odds of selecting the correct answer in respondents who were overweight (OR: 1.18; 95% CI: 0.83–1.69) and obese (OR: 0.90, 95% CI: 0.38–2.14) were not significantly different from the odds for those possessing a normal weight. The majority (79.0%; percentage of correct responses out of all responses adjusted for sampling weights regardless of BMI classification) of respondents were able to identify protein as the type of food to consume to build or maintain muscle. The odds of choosing the correct answer in overweight (OR: 1.06; 95% CI: 0.75–1.50) and obese (OR: 1.08; 95% CI: 0.74–1.59) respondents were not significantly different from the odds for those possessing a normal weight. 

**Figure 1:**
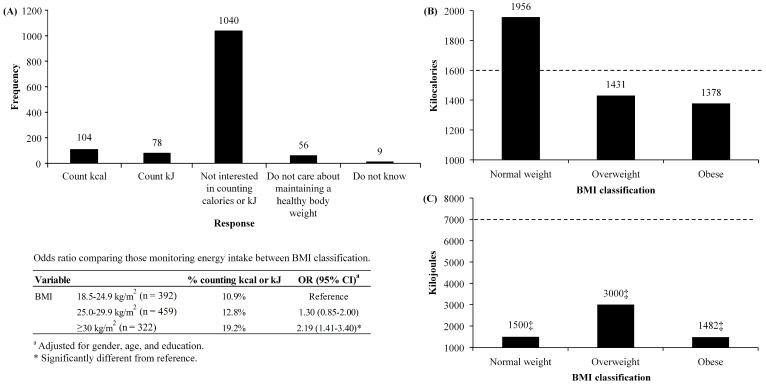
(**A**) What respondents do to maintain a healthy body weight; (**B**) How many kilocalories each respondent thought they needed to consume to maintain their current body weight, data expressed as median values (*n* = 59; adjusted for sampling weights); (**C**) How many kilojoules each respondent thought they needed to consume to maintain their current body weight, data expressed as median values (*n* = 46; adjusted for sampling weights). ---- Represents how many kilojoules should be consumed per day to maintain current body weight of respondents; resting metabolic rate was estimated using the Mifflin-St Jeor equation. ^‡^ = Significantly lower than the kilojoules required to maintain current body weight. Resting metabolic rate: males = 9.99 * weight (kg) + 6.25 height (cm)—4.92 * age (years) + 5; females = 9.99 * weight (kg) + 6.25 height (cm)—4.92 * age (years)—161.

Figure 1A shows the responses to the question about how to maintain a healthy body weight. The majority (80.2%; percentage of responses adjusted for sampling weights regardless of BMI classification) of respondents answered they were not interested in counting kilocalories or kilojoules. The odds of counting kilocalories or kilojoules for obese respondents (OR: 2.19; 95% CI: 1.41–3.40) was significantly higher than the odds for those with normal weight. Figure 1B shows the responses to the question regarding how many kilocalories each respondent should consume to maintain their current body weight according to BMI classification. No significant difference in kilocalories was reported across the groups, H(2) = 1.490, Monte Carlo (with 10,000 iterations) *p* = 0.48. The one-sample Wilcoxon signed-rank test showed the median kilocalories reported from each of the three BMI classification was not significantly different from the calculated value of 1600 kilocalories to maintain current body weight (normal weight: *N* = 12, median = 1956 kilocalories, *p* = 0.162; overweight: *N* = 19, median = 1431 kilocalories; *p* = 0.767; obese: *N* = 28, median = 1378 kilocalories, *p* = 0.304). Figure 1C shows the responses to the question regarding how many kilojoules each respondent should consume to maintain their current body weight by BMI classification. No significant difference in kilojoules was reported across the three groups, H(2) = 4.293, Monte Carlo (with 10,000 iterations) *p* = 0.12. The median kilojoules reported from each of the three groups was significantly lower than the calculated value of 7000 kilojoules to maintain current body weight as shown by the one-sample Wilcox signed-rank test (normal weight: *N* = 15, median = 1500 kilojoules, *p* < 0.001; overweight: *N* = 18, median = 3000 kilojoules; *p* < 0.001; obese: *N* = 13, median = 1482 kilojoules, *p* = 0.01).

### 3.2. Nutritional Behaviors

**Figure 2:**
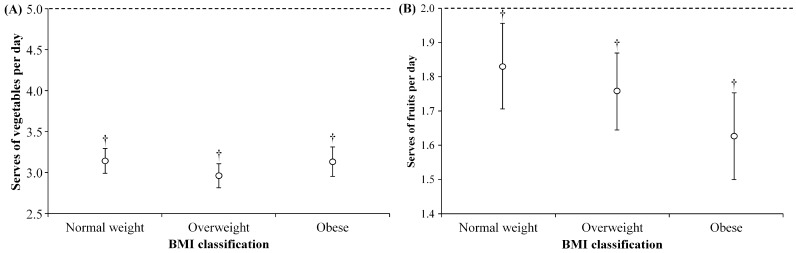
Self-reported servings of (**A**) vegetables and (**B**) fruits consumed per day expressed as mean ± 95% confidence interval. ---- Represents the Australian Government recommendation of daily serves of fruits. ^†^ Significantly lower than the government recommendation, *p* < 0.05.

Figure 2A shows the responses to the question about vegetable consumption relative to BMI classification. There was no significant difference in daily servings of vegetables consumed between BMI classifications, F (2, 1167) = 1.78, *p* = 0.17: normal weight (3.14 ± 1.54), overweight (2.96 ± 1.59), and obese (3.13 ± 1.63). Furthermore, respondents in each BMI classification ate significantly less than the recommended five servings of vegetables per day (*p* < 0.05). Figure 2B shows the responses to the question about fruit consumption by BMI classification. There was no significant difference in daily servings of fruit consumed between BMI classifications, F (2, 1166) = 2.28, *p* = 0.10: normal weight (1.83 ± 1.25), overweight (1.76 ± 1.24), and obese (1.63 ± 1.17). Furthermore, respondents of each BMI classification ate significantly less than the recommended two servings of fruit per day (*p* < 0.05). The results of the ANOVA in regard to the consumption of daily serves of vegetables and fruits held true after adjusting for the covariates of gender, age, and education using separate multiple linear regression analyses. 

When pooling the data, 50.5% (percentage of responses adjusted for sampling weights regardless of BMI classification) of the respondents answered they had eaten fast food during the previous week. Further, the odds of eating fast food for overweight (OR: 1.52; 95% CI: 1.13–2.04) and obese (OR: 1.43; 95% CI: 1.04–1.98) were significantly higher than the odds for those with normal weight (Table 4).

## 4. Discussion

Overweight/obesity presents significant issues regarding the sustainability of the current healthcare system along with quality and duration of life in overweight/obese individuals. As a result, public health initiatives have been implemented in an attempt to promote healthy body weight, but with increasing prevalence of overweight/obesity in Australia [3] the argument can be made these initiatives have been ineffective [2,24]. Regardless, many have argued increasing nutritional knowledge is required to improve nutritional behaviors [9,10,11,12,13], and therefore we sought to examine if differences exist in nutritional knowledge and eating behaviors in normal, overweight, and obese adults residing in Central Queensland, Australia. Results from our investigation support findings from previous investigations conducted in Europe [16,17] and America [19] which concluded lack of nutritional knowledge does not appear to be the primary cause of obesity.

In regard to nutritional knowledge, our work is in agreement with Hendrie et al. [15] who reported residents of Adelaide, South Australia understood basic nutritional knowledge but struggled with applying nutritional knowledge. Results from our investigation found residents had a good understanding of basic nutritional knowledge with no differences existing between BMI classifications for the identification of saturated fat as an unhealthy fat and the use of protein to build/maintain muscle. However, knowledge was lacking in the area of daily energy requirements to maintain current body weight in the form of kilocalories and kilojoules; which can be argued is the most important nutritional knowledge question to understand in regards to weight management. Specifically, in our investigation, 95% of respondents chose not to answer the question regarding the amount of kilocalories required to maintain their current body weight and 96% of respondents chose not to answer the question regarding the amount of kilojoules required to maintain their current body weight. The response rate for the other nutritional knowledge questions was 100% (although not all responses were included as some residents did not provide information about height or weight and could not be placed in a BMI classification), suggesting respondents may not have answered the questions regarding the amount of kilocalories and kilojoules required to maintain their current body weight because they did not know the answer.

For residents who answered the questions regarding the amount of kilocalories and kilojoules required to maintain their current body weight it became apparent that a better understanding exists in regard to kilocalories than kilojoules. Each of the BMI classifications were able to correctly identify the amount of kilocalories required to maintain current body weight, while none of the BMI classifications were able to correctly identify the amount of kilojoules required to maintain their current body weight. Furthermore, each of the BMI classifications grossly underestimated the amount of kilojoules required to maintain body weight, providing estimates closer to the amount of kilocalories required to maintain their current body weight. To our knowledge this is the first study to directly compare knowledge of appropriate kilocalorie and kilojoule intake to maintain body weight and our findings support previous research suggesting energy in food is a difficult concept for the general public to understand [25]. It is also important to note kilojoules are the primary energy measurement used on Australian food packaging [26] but residents have a better understanding of kilocalories; this finding should be considered for future food packaging guidelines in Australia.

We were concerned to find that although 35% of residents were overweight and 25% were obese, 81% were not interested in counting kilocalories or kilojoules, 8% monitored kilocalories, 6% monitored kilojoules, and 4% did not care about maintaining a healthy bodyweight. Given the prevalence of overweight/obesity in Central Queensland and the laissez faire attitude towards monitoring energy consumption, it was no surprise to find each of the BMI classifications consumed less than the daily recommended servings of vegetables and fruits. The under-consumption of vegetables and fruit is consistent with national data which reported vegetable and fruit intake has been decreasing since 2001, with 6% of Australian adults consuming the recommended daily serves of vegetables and fruit [2].

We also found 51% of residents had eaten fast food during the previous week with the highest frequency of fast food consumption occurring in overweight and obese residents. However, fast food consumption was high across all BMI classifications (normal weight: 48%, overweight: 54%, obese: 51%) and is likely a primary contributor to the excess energy consumption among residents of Central Queensland. Specifically, the most recent Australian Dietary Guidelines suggest minimizing consumption of discretionary food [27] but the latest survey of Australians’ food habits reported 35% of total daily energy consumption was from discretionary foods [28]. 

Previous research conducted on a national level in Australia reported nutritional messages were being received by the population but not put into practice [24]. This finding is congruent with the theory proposed by Story et al. [29] that nutritional knowledge is not the primary factor influencing the prevalence of overweight/obesity but rather food and eating environments. Results from our work support the work of others who reported lack of knowledge is unlikely the primary cause of overweight/obesity [16,17]. We do not dispute the importance of nutritional education; however, we support the suggestion of others who proposed nutritional education alone is typically unable to facilitate behavior change as personal, behavioral, and environmental factors are overlooked [16,18,30,31]. Future research should utilize longitudinal experimental designs to determine optimal intervention strategies to help residents maintain a healthy body weight.

## 5. Limitations

Several study limitations should be noted. First, due to the cost of administering the survey we were limited in the number of questions we could ask respondents. However, each question is ecologically valid and can easily be replicated in future research. Furthermore, our study is not intended to be the final work in the area but is intended to provide an initial line of inquiry and promote future work in the area. Second, respondents were classified as normal weight, overweight, or obese by self-reported height and weight which were used to calculate BMI. BMI does not reflect body fat distribution or the same degree of fatness across different individuals. However, at the population level, BMI has been identified as a practical and useful measure for the classification of overweight and obese individuals [32]. Third, we did not account for physical activity in our questions that assessed how many kilocalories and kilojoules each respondent thought they needed to consume to maintain their current body weight. However, if we were to account for the physical activity level of respondents it would increase the amount of energy they would be required to consume per day to maintain their current body weight. Normal weight, overweight, and obese respondents correctly identified the amount of kilocalories required to maintain their current body weight. Importantly, normal weight, overweight, and obese respondents all significantly underestimated the amount of kilojoules required to maintain their current body weight. As a result, our data demonstrate that respondents underestimated the amount of daily energy in the form of kilojoules they should consume per day to maintain their current body weight. Therefore, not accounting for the physical activity level of respondents was not the reason for respondent inaccuracy to this question.

## 6. Conclusions

Meeting recommended nutritional guidelines has numerous health benefits including maintenance of healthy body weight and reduced risk of developing chronic disease [27]. The knowledge deficit model is commonly used to explain the cause of obesity [9,10,11,12,13] but research suggests factors other than knowledge contribute to overweight/obesity [16,17,18,29,30]. The primary findings from our study were: (1) Independent of BMI, residents had a good understanding of general nutritional knowledge related to macronutrients; (2) Independent of BMI, residents had a better understanding of kilocalories than kilojoules required to maintain current body weight. This finding is concerning as kilojoules are the primary unit of energy measurement on Australian food packaging, but our results suggest that residents have a better understanding of kilocalories; (3) Despite finding residents have an understanding of general nutritional knowledge, independent of BMI, residents chose to consume unhealthy diets characterized by low vegetable and fruit consumption combined with regular consumption of fast food.

## Figures and Tables

**Table 1:** Nutritional knowledge and nutritional behavior questions asked to respondents.

Question	Answer Choice(s)
**Nutritional Knowledge**	
Which of these fats is a poor food choice?	Saturated fat, unsaturated fat, Omega-3 fatty acids, don’t know/unsure
Which is a good choice to build or maintain muscle?	Protein (steak, chicken, fish), carbohydrate (potato, apple, orange), fat (cookies, ice cream), don’t know/unsure
How many calories should you consume per day to maintain your current body weight?	Open answer
How many kilojoules should you consume per day to maintain your current body weight?	Open answer
**Nutritional Behavior**	
How many servings of vegetable do you eat on a usual day?	Open answer
How many servings of fruit do you eat on a usual day?	Open answer
In the last week did you consume fast food?	Yes or no
What do you do to maintain a healthy body weight?	Count kilocalories, count kilojoules, not interested in counting kilocalories or kilojoules, do not care about maintaining a healthy body weight

**Table 2:** Descriptive information of survey respondents.

Variable	Categories	%
Gender	Male	64.6
	Female	35.4
Age	18–34 years	30.9
	35–44 years	19.6
	45–54 years	18.2
	55+ years	31.4
Body Mass Index	<18.5 kg/m^2^	1.7
	18.5–24.9 kg/m^2^	30.2
	25.0–29.9 kg/m^2^	35.4
	≥30 kg/m^2^	24.9
	No response	7.8
Household Income	Up to $26,000	12.5
	$26,001–$52,000	11.5
	$52,001–$100,000	17.9
	>$100,000	25.9
	Don’t know/No response	32.2
Years of Education	1–10 years	28.6
	11–12 years	25.9
	13–14 years	16.1
	15+ years	28.9
	No schooling	0.1
	Don’t know/No response	0.4

Note: *N* for survey sample = 1289; data were adjusted for sampling weights.

**Table 3:** Nutritional knowledge between body mass index classifications in rural and regional Central Queensland residents.

BMI Classification		% Correct	OR (95% CI) *
	**Ability to Choose the Correct Answer Regarding Types of Dietary Fats**		
Normal	18.5–24.9 kg/m^2^ (*N* = 392)	73.5%	Reference
Overweight	25.0–29.9 kg/m^2^ (*N* = 459)	71.9%	1.18 (0.83–1.69)
Obese	≥30 kg/m^2^ (*N* = 322)	77.0%	0.90 (0.38–2.14)
	**Ability to Choose the Correct Answer Regarding Protein in the Diet**		
Normal	18.5–24.9 kg/m^2^ (*N* = 392)	80.4%	Reference
Overweight	25.0–29.9 kg/m^2^ (*N* = 459)	78.9%	1.06 (0.75–1.50)
Obese	≥30 kg/m^2^ (*N* = 322)	78.9%	1.08 (0.74–1.59)

* Adjusted for gender, age, and education.

**Table 4:** Odds ratio comparing fast food consumption between BMI classifications in rural and regional Central Queensland residents.

BMI Classification		% Eating Fast Food	OR (95% CI) *
Normal	18.5–24.9 kg/m^2^ (*N* = 391)	48.3%	Reference
Overweight	25.0–29.9 kg/m^2^ (*N* = 460)	53.7%	1.52 (1.13–2.04) ^#^
Obese	≥30 kg/m^2^ (*N* = 322)	50.9%	1.43 (1.04–1.98) ^#^

* Adjusted for gender, age, and education. ^#^ Significantly different from reference.
